# A Neuron-Like Cellular Model for Severe Tinnitus Associated with Rare Variations in the *ANK2* Gene

**DOI:** 10.1007/s12035-024-04674-8

**Published:** 2025-01-15

**Authors:** Mar Lamolda, Lidia Frejo, Juan Martin-Lagos, Francisca E. Cara, Alvaro Gallego-Martinez, Jose A. Lopez-Escamez

**Affiliations:** 1 Otology & Neurotology Group CTS495, Division of Otolaryngology, Department of Surgery, Instituto de Investigación Biosanitaria, Ibs.GRANADA, Granada, Universidad de Granada, Granada, Spain; 2 Sensorineural Pathology Programme, Centro de Investigación Biomédica en Red en Enfermedades Raras, CIBERER, Madrid, Spain; 3 Meniere’s Disease Neuroscience Research Program, Faculty of Medicine & Health, School of Medical Sciences, The Kolling Institute, University of Sydney, 10 Westbourne St, St Leonards, Sydney, NSW Australia; 4 Department of Otorhinolaryngology, Hospital Clinico Universitario San Cecilio, Granada, Spain

**Keywords:** Tinnitus, HiPSC, Stem cells, Disease model, Inner ear neurons, Meniere disease

## Abstract

Tinnitus is the perception of sound without an external source, often associated with changes in the auditory pathway and different brain regions. Recent research revealed an overload of missense variants in the *ANK2* gene in individuals with severe tinnitus. *ANK2*, encoding ankyrin-B, regulates axon branching and inhibits microtubule invasion. Missense mutations in ANK2 may promote excessive axonal branching and the formation of excitatory synapses. This study aims to generate a patient-derived iPSC model from an individual with severe tinnitus and to differentiate these cells into otic-neural progenitors and inner ear neurons. We successfully generated a severe tinnitus cellular model through cell reprogramming. Using a two-stage neural differentiation protocol, we differentiated these cells into otic-neural progenitors and neuron-like cells. We confirmed the expression of genes, proteins, and cellular markers, including *ANK2*, otic-neural progenitors, and neuron-like cells through qPCR and immunostaining. Our analysis revealed higher *ANK2* expression in the control cell line compared to the patient cell line. Although both lines formed multipolar neurons, the patient cell line displayed a unique pattern of closely grouped neurons with increased neuronal projections and dendrites compared to the control. This cellular model provides a valuable tool for studying the cellular and molecular changes associated with the *ANK2* gene. It holds great promise for the development of novel drug and gene-based therapies for severe tinnitus.

**Supplementary Information:**

The online version contains supplementary material available at 10.1007/s12035-024-04674-8.

## Introduction

Tinnitus is the perception of a phantom sound without any external source, affecting between 10 and 15% of the general population. Of these, 1–3% can be diagnosed with a debilitating tinnitus disorder associated with sleep disturbances and psychological distress, leading to significant emotional, behavioral, and health-related quality-of-life impacts [1]. Despite the high burden on healthcare systems, treatments for tinnitus are presently lacking. This is partly due to the heterogeneity of tinnitus presentation, which makes the development of universal therapies challenging [2].

Tinnitus is not only a symptom associated with hearing loss; it is also considered the result of exacerbated plasticity of the central auditory system in response to the crosstalk with auditory nerve fibers [3]. Its development is related to different nuclei of the auditory pathway, particularly the cochlear nuclei and the primary auditory cortex. Additionally, tinnitus perception involves multiple brain areas and neural networks, such as the hippocampus or prefrontal cortex [4]. Likewise, it can involve unknown auditory and non-auditory networks and molecular pathways. This complexity has slowed progress in the field, with recent research focusing on the genetic contributions to tinnitus.

Epidemiological studies in twins and adoptees indicate a strong genetic component to tinnitus, particularly for severe and bilateral cases, with evidence of familial aggregation and higher susceptibility in women [5]. Genome-wide association studies (GWAS) have reported few significant genetic associations (*p* < 10^−8^), particularly among individuals with noise exposure [6, 7], but most of these findings are awaiting replication in larger cohorts.

Recently, exome sequencing has been used to identify rare genetic variants in individuals with extreme tinnitus phenotypes (EP) [8]. This approach has led to the identification of the *ANK2* gene as a potential candidate, linking membrane trafficking and cytoskeletal protein binding to the pathophysiology of severe tinnitus [9].

*ANK2* encodes ankyrin-B, a scaffolding protein that anchors the axonal plasma membrane to L1CAM, playing a crucial role in the development, maintenance, and refinement of neural circuits across different brain regions. Ankyrin-B suppresses axon branching by coordinating cortical microtubules (MTs) and preventing MT invasion into new axon branches through direct interaction with MTs. Therefore, deficiencies or mutations in the *ANK2* gene may exacerbate axonal branching, resulting in ectopic neuronal connections and increased excitatory synapses. This branching mechanism may enhance connectivity between auditory and non-auditory regions, particularly the para-hippocampus, contributing to the severity of tinnitus [9–11].

Disease modeling using patient-derived induced pluripotent stem cells (iPSC) offers a valuable approach to studying rare neurological diseases. The goal of this study was to generate an iPSC line from a severe tinnitus patient carrying mutations in the **ANK2** gene. These iPSCs were then differentiated into neuronal progenitors and neuron-like cells to assess how this rare variant affects cellular phenotype.

## Materials and Methods

### Patient Selection

A female patient with chronic persistent tinnitus and a rare variant in the ANK2 gene was selected from the Meniere disease (MD) Consortium database [12]. She has suffered MD and tinnitus for 50 years and reported severe distress according to the tinnitus handicap inventory (THI) score of 76. After providing detailed information about the study’s goal, she gave informed consent to participate and donated a blood sample to generate the cell line. The Granada Ethical Review Board for Clinical Research approved the research protocol.

### Psychoacoustic Characterization

An online tone generator (http://www.onlinetonegenerator.com) was used to find the most similar tinnitus and to characterize its loudness and frequency. The process began by introducing pure-tone sounds, starting at 1000 Hz, with the frequency adjusted—either ascending or descending—based on the patient’s feedback, until the perceived tinnitus tone was matched (frequency range: 125–16,000 Hz). If the patient did not recognize their tinnitus as a pure tone, we presented different noise types (white, brown, or pink) until the patient identified the most similar sound. Following this, acufenometry was conducted in a soundproof booth to verify the noise type or frequency determined through the tone generator. During this process, the tinnitus loudness (in dB SL) and the minimum masking level (MML) were measured. Additionally, residual inhibition was assessed in the affected ear, which was considered positive if there was a reduction in intensity or complete disappearance of the tinnitus for at least 20 s. The audiological assessment was conducted using an AC40 clinical audiometer (Interacoustics, Middelfart, Denmark).

### Psychometric Characterization

Two questionnaires, the tinnitus handicap inventory (THI) and the visual analog scale (VAS), were used to assess tinnitus-related discomfort and measure tinnitus’s impact on the patient’s quality of life. In addition, two standardized depression and anxiety questionnaires, the patient health questionnaire (PHQ-9) and the hospital anxiety and depression scale (HADS), were used. Finally, the Montreal cognitive assessment (MoCA) was used to evaluate possible mild cognitive dysfunction.

### Cell Derivation and Culture

The PBMC1-iPS4F1 healthy cell line generation and cell characterization was derived from a Spanish female and published elsewhere [13]. The patient’s hiPSC line, ANK2-24, was obtained by reprogramming peripheral blood mononuclear cells (PBMCs) and transducing them with non-integrative Sendai virus (SeV) vectors containing the reprogramming factors *OCT3/4*, *SOX2*, *KLF4*, and *c-MYC* (CytoTune-iPS 2.0 Sendai Reprogramming kit, ThermoFisher Scientific). Both hiPSC lines were maintained in mTeSR1 medium (STEMCELL Technologies) at 37 °C and 5% CO_2_ and passaged when needed.

### hiPSCs Differentiation into Inner Ear Neurons

We used a neural differentiation protocol based on Boddy et al. [14] to generate inner ear neurons (IENs). The differentiation protocol consisted of two phases: phase 1 involved the generation of otic neuronal progenitors (ONPs), and phase 2 involved the formation of IENs.

#### Generation of ONPs Derived from hiPSCs (Phase 1)

To differentiate hiPSCs into ONPs, hiPSCs were plated at a density of 8000 cells/cm^2^ in DFNB medium (DMEM/F12 with Glutamax (Gibco, UK) supplemented with 1 × N2 and 1 × B27 (Gibco, USA) supplemented with 50-ng/ml FGF3 (Palex Medical, Spain), 50-ng/ml FGF10 and 10 uM IWR-1 (R & D Systems, UK) onto laminin-coated 6-well culture plates. The cell culture media was changed every other day until day 8. On day 9, IWR-1 was replaced by 2 uM BIO (Merck, Germany) while maintaining FGF3 and FGF10 until day 12, with media changing every other day. After phase 1, ONPs were cultured in DFNB supplemented with 20-ng/ml bFGF, 50-ng/ml IGF (Peprotech, UK), and 20-ng/ml EGF (R & D Systems, UK) at a density of 20,000 cells/cm^2^.

#### Cell Doubling Time and Cell Viability Assays

We performed cell doubling time and cell proliferation assays to differentiate ONPs into IENs in a suitable range of culture passages. Cell doubling time was achieved by plating 4000 cells/cm^2^ at day 0 onto 0.2% gelatin-coated 12-well culture plates. Cell numbers were determined by counting in a Neubauer chamber daily for 4 days. Moreover, cell viability assays were performed using CellTiter96 Aqueous One Solution Reagent (Promega, USA), a colorimetric method to determine the number of viable cells in proliferation. ONPs were plated at 10,000 cells/cm^2^ density at day 0 on a 96-well culture plate previously coated with gelatin. On day 4, the CellTiter96 reagent was added to the cell culture, and at 3 h, the cell viability was determined by absorbance in an Infinite M200 Nanoquant reader (Tecan, Switzerland).

#### Generation of Sensory Inner Ear Neurons from hiPSC-Derived Otic Neuronal Progenitors (Phase 2)

ONP cultures derived from hiPSCs were dissociated using 1:10 trypsin solution (Sigma-Aldrich, UK) and plated at a density of 4000 cells/cm^2^ onto gelatin-coated 6-well culture plates in DFNB medium supplemented with 20-ng/ml bFGF (PeproTech, UK) and 500-ng/ml human Shh-C24II (Miltenyi Biotec, Spain). The cell culture medium was replaced every other day. From day 7 forward, Shh-C24II and bFGF were removed from the cell culture medium. RNA and protein were isolated at days 0, 7, 14, and 21. were fixed for immunofluorescence staining at days 14 and 21.

### Quantitative Real-Time PCR

We used the High Pure RNA Isolation Kit (Roche, Switzerland) to isolate total RNA from cell lysates, according to the manufacturer’s protocol. Then, we used the Maxima’s first strand cDNA synthesis kit for RT-qPCR with dsDNase (Thermo Fisher Scientific, US). Q-PCR was run using Sybr Green (Quantabio, MA, USA) and 10 ng/uL of total RNA per reaction in the QuantStudio 6 Real-Time PCR thermocycler (ThermoFisher Scientific, Waltham, MA, USA). Relative quantification was performed using GAPDH as the housekeeping gene. Fold changes for each gene were calculated using the 2^−ΔΔCT^ method against the day 0 undifferentiated ONP. All primers (Sigma Aldrich, USA) used are reported in Supplementary Table 1.

### Immunofluorescence Staining

Cells were fixed with 4% paraformaldehyde (Sigma Aldrich, USA) in 1 × phosphate-buffered saline (PBS) for 20 min at room temperature. Then, permeabilized and blocked with 0.3% Triton-X (Sigma Aldrich, USA), and 3% bovine serum albumin (BSA; Sigma Aldrich, USA) in PBS for 30 min. Cells were then incubated with primary antibodies in 3% BSA in PBS overnight at 4 °C. Secondary antibodies were added in 10% BSA in PBS and incubated for 1 h at room temperature in the dark. Subsequently, the nuclei were stained with 1-μg/mL Hoechst (Thermo Fisher Scientific, USA) for 5 min. Antibodies (all Thermo Fisher Scientific) and dilutions used in this study are reported in Supplementary Table 2. Cells were imaged on a Zeiss LSM 710 confocal microscope (Zeiss, Germany). All images were captured using identical settings for the posterior quantification. ImageJ (NIH) software [15] was used for image processing and analysis. Negative controls were determined by omitting the primary antibodies during the immunofluorescence. ImageJ software was used to determine the quantification of ANK2 by counting cells with characteristic morphologies at days 14 and 21 of neuronal differentiation. Results are reported as mean fluorescence intensity/cell of 100 total cells ± SEM.

### Western Blot Analysis

For total protein extraction, sensory inner ear neuron cultures from hiPSCs-derived ONPs from day 0 to day 21 were lysed using cell lysis buffer 10 × (Cell Signaling Technology, USA) containing protease inhibitor cocktail (Sigma Aldrich, USA). Cell lysates were separated by molecular weight using precast polyacrylamide gels (Bio-Rad, USA) and transferred to nitrocellulose membranes (Bio-Rad, USA). Proteins were detected using the ChemiDoc XRS + System (Bio-Rad, USA). To detect ANK2, the ankyrin B polyclonal antibody (PA5-82,326, Invitrogen) was used. GAPDH (SAB3500247, Sigma Aldrich) loading control for total protein extractions was used. Western blotting was carried out using standard procedures. Image processing and quantification of band intensity were executed using Image Lab software (Bio-Rad, USA).

### Statistical Analysis

We used a two-way ANOVA statistical comparison and Sidak’s multiple comparisons test across the different cell lines using a *p* value < 0.05 as a threshold to test the expression of otic and neural genes. We used a one-way ANOVA statistical comparison and Sidak’s multiple comparisons test across the control and patient line using a *p* value < 0.05 as a threshold to analyze the expression of ANK2 in confocal images and western blot. The graphics and the statistical tests were run in GraphPad Prism software version 9.0 (San Diego, CA).

## Results

### Patient Selection and Audiological Characterization

A Spanish female patient was diagnosed with definite MD. She has experienced severe tinnitus in her left ear for over 30 years, and recent exome sequencing (published elsewhere) identified a mutation in the *ANK2* gene (Missense variant in 4:114,294,537 G/A).

**Fig. 1:**
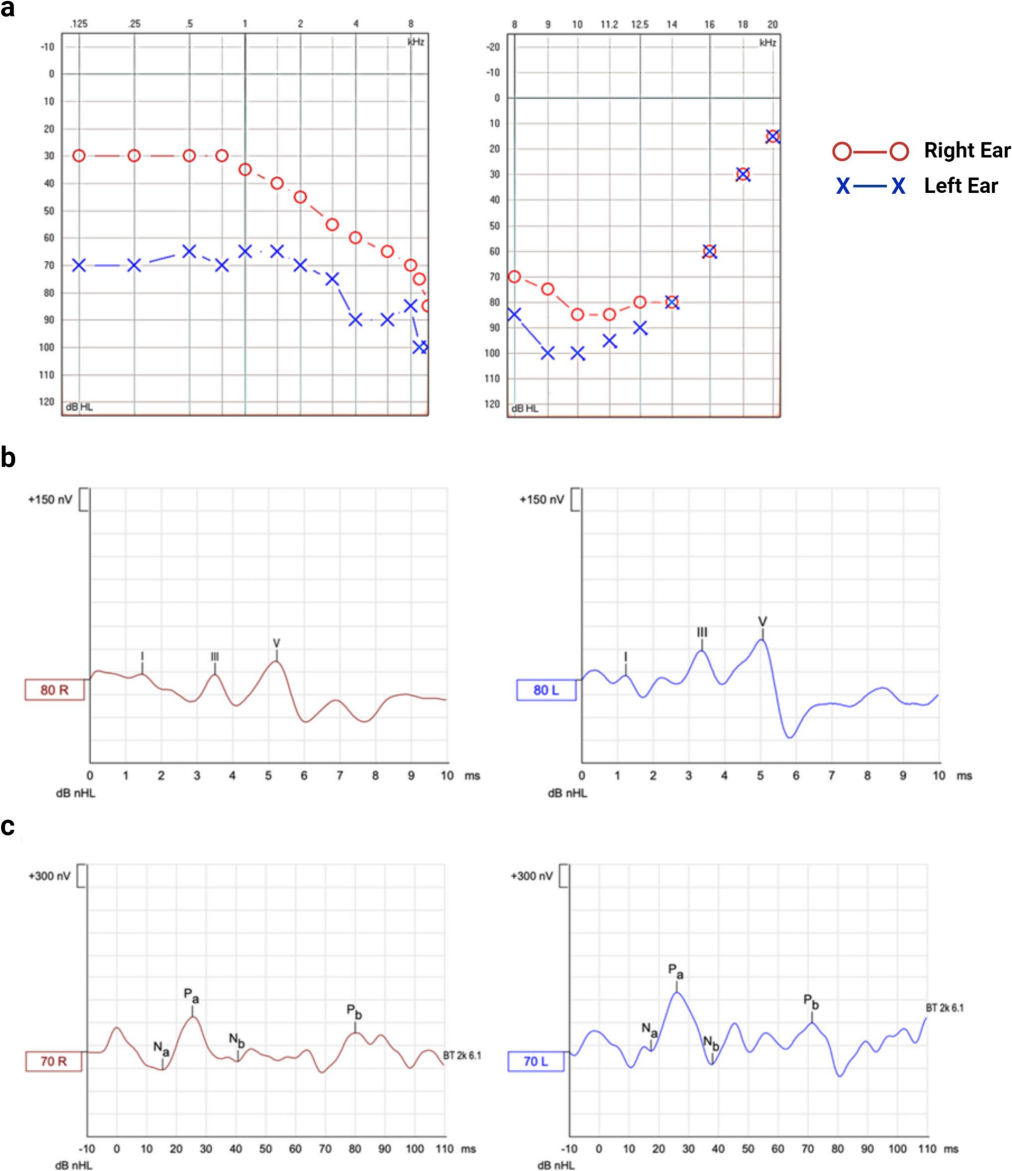
**a** Pure-tone audiometry (left) showing hearing thresholds (dB HL) for right ear (red dots) and left ear (blue crosses) in the frequency spectrum from 125 to 8000 Hz (0.125–8 kHz); high-frequency pure-tone audiometry (right) showing hearing thresholds (dB HL) for right ear (red dots) and left ear (blue crosses) in the frequency spectrum from 8000 to 20,000 Hz (8–20 kHz). **b** Recording of auditory brainstem responses (ABR) (above) evoked at 80 dB in both ears (red line, right ear; blue line, left ear) where the different components (I, III, and V waves) with their corresponding latencies (ms) and amplitudes (nV) can be identified (1 µV = 1000 nV). **c** Recording of auditory middle latency responses (AMRL) (below) evoked at 70 dB in both ears (red line, right ear; blue line, left ear) where the different components (Na, Pa, Nb, and Pb waves) with their corresponding latencies (ms) and amplitudes (nV) can be identified (1 µV = 1000 nV)

Pure-tone audiometry revealed bilateral SNHL, with moderate hearing loss in the right ear and severe loss in the left ear. Both ears showed profound hearing loss at high frequencies (Fig. 1). Acufenometry showed pitch-matching tinnitus as white noise, with a threshold of 75dBLH, a tinnitus loudness + 3dBSL, and MML + 9dBSL. Partial residual inhibition was also observed.

The patient’s psychometric profile was as follows: *THI*: 96 (severe disability); *VAS*: 9 out of 10; *PHQ-9*: 14 out of 27 (moderate depression); *HADS-Anxiety*: 19 out of 21 (case of anxiety): *HADS-Depression*: 13 out of 21 (case of depression); *MoCA*: 22 out of 30 (indications of mild cognitive impairment).

The latencies and amplitudes of ABR and AMLR at 70dBnHL and 50dBnHL, respectively, are shown in Table 1. The amplitudes of ABR waves I, III, and V were higher in the tinnitus-affected ear, while the latencies of these waves were lower in the unaffected ear. The latencies of the AMLR components varied from one ear to the other without a consistent pattern. However, the amplitudes of the positive AMLR waves (Pa and Pb) were greater in the left (tinnitus-affected) ear compared to the contralateral ear.

**Table 1:** ABR and AMLR latencies and amplitudes

**Wave**	**Latency (ms)**		**Amplitude (µV**)	
	Right	Left	Right	Left
**ABR**				
**I**	1.50	1.23	0.112	0.142
**III**	3.53	3.37	0.202	0.210
**V**	5.23	5.07	0.402	0.646
**AMLR**				
**Na**	15.33	18.01	0.240	0.070
**Pa**	25.67	26.16	0.456	0.695
**Nb**	40.33	37.67	0.140	0.260
**Pb**	80.67	71.67	0.243	0.291

### ANK2-24 Cell Line Characterization

Cell characterization of the ANK2-24 line showed typical pluripotent cell morphology and expresses pluripotency markers such as SSEA4, SSEA3, TRA1-81, TRA1-60, and endogenous pluripotency genes like *NANOG*, *SOX2*, and *OCT3/4*. It presented a normal karyotype (46, XX), and pluripotency was shown by differentiation into the three germ layers of an embryo (Supplementary Fig. 1).

### Generation of Inner Ear Neurons from the ANK2-24 Cell Line

#### ANK2-24 Differentiation into IENs

**Fig. 2:**
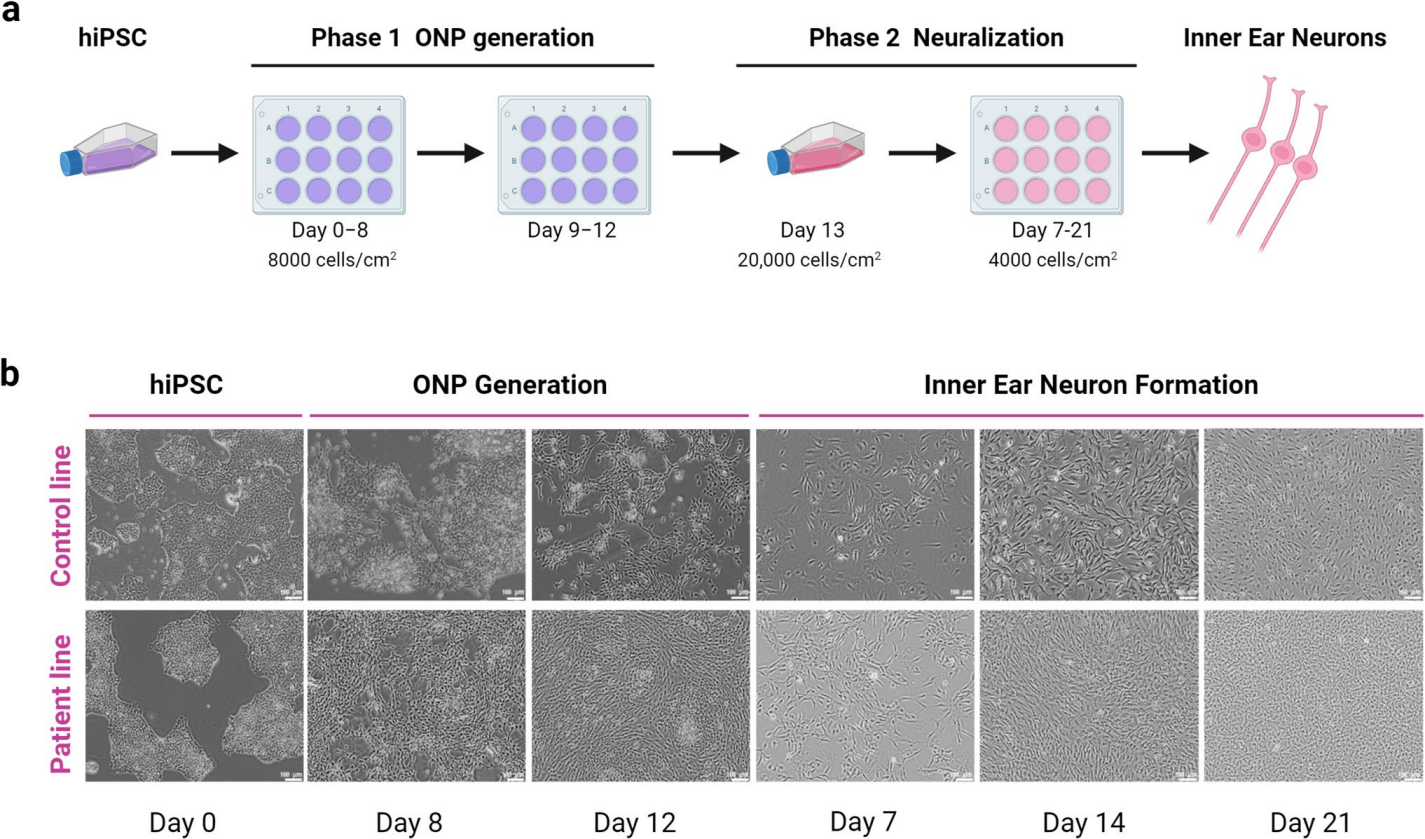
Generation of IENs derived from hiPSC. **a** Schematic representation of neural differentiation protocol from hiPSC for 21 days. **b** Images of the IEN formation were obtained by optic microscopy for 21 days

The ANK2-24 line was differentiated to IENs using a two-step neuronal protocol. The first step was to generate ONPs. The second step was the neuralization of ONPs into IENs. The hiPSC control line and ANK2-24 line were cultured for 12 days in an otic-neural induction medium. Then, the ONPs generated were differentiated into sensory IEN-like cells for 21 days in a neuralization medium. The control and patient hiPSC lines developed ONP-derived IENs after 21 days of culture; cells showed extended neurite projections and neural networks between cells (Fig. 2).

#### Expression of Otic-Neuronal Progenitors

During neural differentiation, PBMC1-iPS4F1 and ANK2-24 cell lines expressed otic markers such as PAX8 and FOXG1 and neural markers such as β-III TUBULIN (TUJ1) and POU4F1 (BRN3A), which are implicated in neurogenesis and ganglion sensory neurons development, respectively. The expression of *ANK2*, *PAX8*, *FOXG1*, and *β-III TUBULIN* was validated by qPCR.

**Fig. 3:**
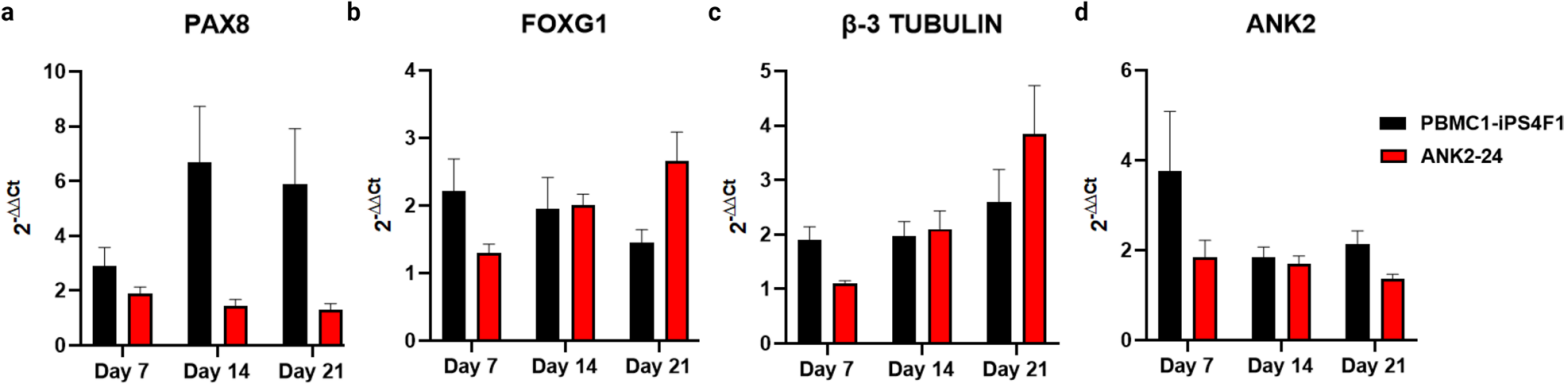
Gene expression of otic-neural progenitors. **a** Expression of PAX8. The PAX8 gene expression is higher in control and patient lines. **b** FOXG1 expression. The FOXG1 gene expression in the patient line is delayed compared to the control line. **c** Expression of β−3 TUBULIN. Both cell lines show an increase of β−3 TUBULIN expression at day 21. **d** Expression of *ANK2* gene. The control cell line presents a higher expression of ANK2 when compared to the patient line

The levels of *PAX8* expression were higher in the control line than in the patient line, decreasing its expression on day 21 (Fig. 3a). The expression of FOXG1 in the PBMC1-iPS4F1 line decreased until day 21. However, the ANK2-24 line showed an increased *FOXG1* expression on day 21 because of a delay in its otic differentiation (Fig. 3b). By contrast, the maturation of IEN was observed in both cell lines, showing an increase of *β-III TUBULIN* expression on day 21 (Fig. 3c). The level of the mutated gene expression *ANK2*, was higher in the control line during all otic-neural differentiation, especially on day 7 (Fig. 3d).

#### Expression of Otic-Neuronal Cell Markers

**Fig. 4:**
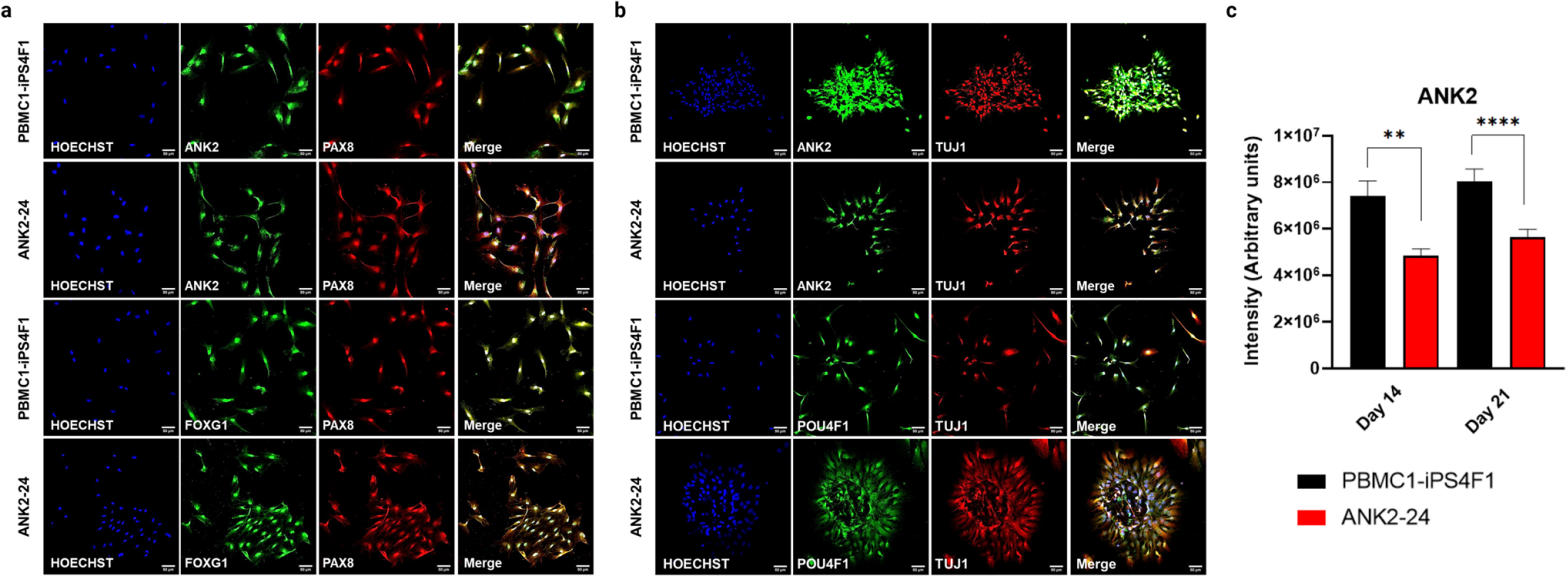
Expression of otic-neural cell markers by immunostaining. **a** Confocal imaging of otic markers expression at day 14 of otic-neural differentiation. Both cell lines express ANK2 (green), PAX8 (red), and FOXG1 (green) markers. **b** Confocal imaging of neural markers expression at day 21 of otic-neuronal differentiation. Both cell lines express ANK2 (green), POU4F1 (green) and TUJ1 (red) markers. **c** *ANK2* gene quantification. The PBMC1-iPS4F1 line shows a higher expression of the *ANK2* than the ANK2-24 cell line (**p < 0.003, ****p < 0.0001)

The expression of the cell markers ANK2, PAX8, FOXG1, POU4F1, and TUJ1 (β−3-TUBULIN) at day 14 and day 21 during IEN generation was validated by immunostaining. On day 14, both cell lines expressed the progenitors’ markers FOXG1 and PAX8. Moreover, cells showed co-expression between ANK2 and PAX8 cell markers and FOXG1 and PAX8 (Fig. 4a). On day 21, cells presented neuronal projections and expressed the neural markers POU4F1 and TUJ1. Both cell lines exhibited a co-expression of ANK2 and TUJ1 and POU4F1 and TUJ1 (Fig. 4b). The ANK2 expression level observed in the confocal images showed a higher expression of ANK2 maker in the control line compared to the patient line. On day 21 of neural differentiation, the ANK2 expression in PBMC1-iPS4F1 and ANK2-24 lines was higher than on day 14. The ANK2 expression was elevated in the control cell line. These results showed significant differences between the control and patient cell lines at both day 14 and day 21, with a *p* value < 0.05 (Fig. 4c).

**Fig. 5:**
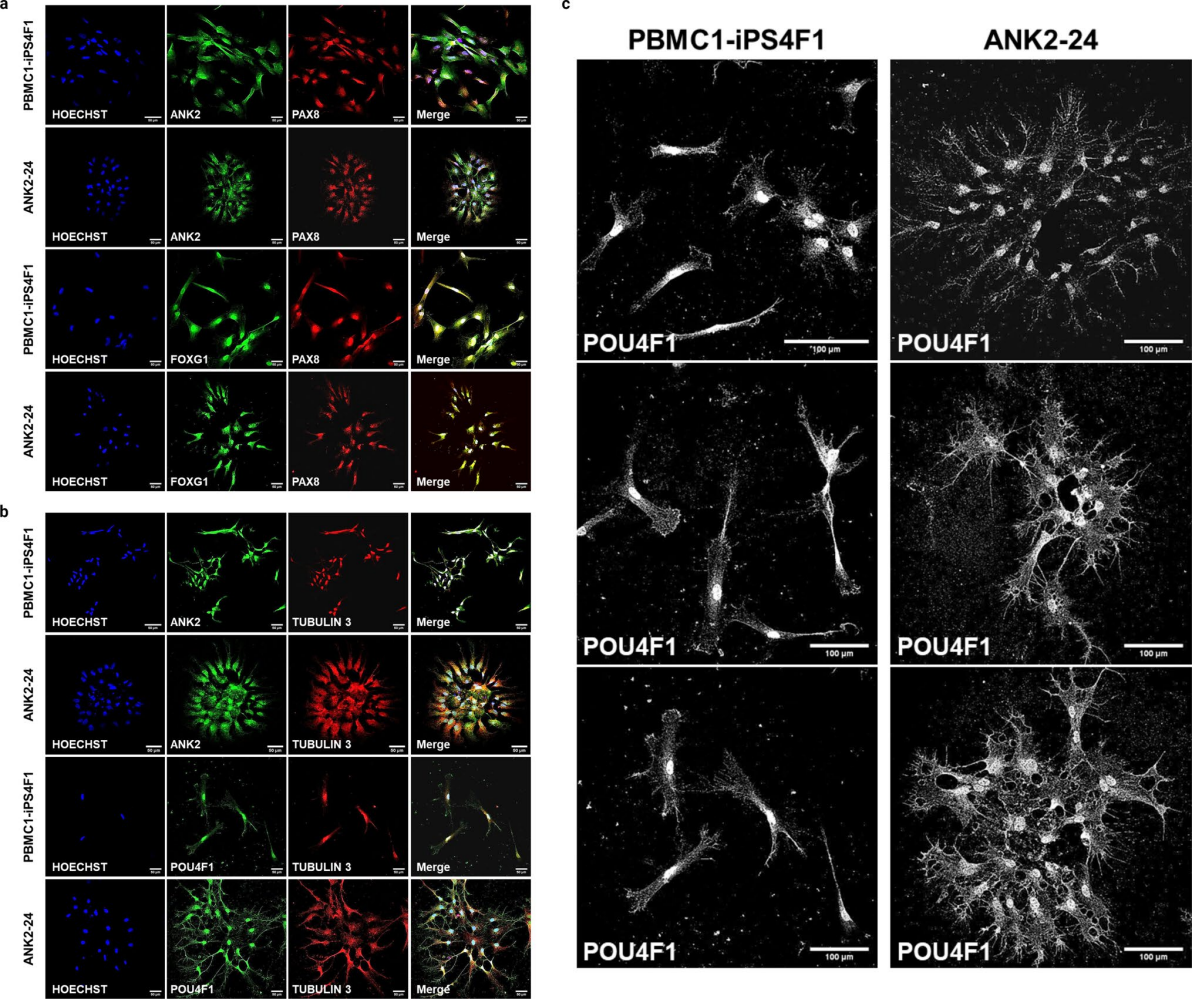
Confocal images of otic-neural differentiation a higher-magnification (40 ×). **a** Expression of otic markers on day 14 of IEN differentiation. **b** Expression of neural markers on day 21 of IEN differentiation. **c** Expression of POU4F1 (grayscale) on day 21 of neural differentiation. The patient cell line displays its IEN-forming clusters with higher neural projections and dendrites than the control cell line

Figure 5 provides a detailed view of IEN markers analyzed through confocal imaging, showing the expression of ANK2, FOXG1, and PAX8 at day 14 in both the control and patient cell lines (Fig. 5a). In contrast, Fig. 5b shows the expression of ANK2, POU4F1, and TUJ1 on day 21 for both cell lines, supporting the earlier description of otic-neural marker expression. Additionally, Fig. 5c provides a detailed, magnified confocal image of POU4F1, a transcription factor involved in specifying inner ear sensory neurons and guiding neurite projections. This allows for a more detailed analysis of the morphology of neuronal projections in both control and patient cell lines during neural differentiation.

Both cell lines show the presence of multipolar neurons with more than two neural projections and several dendrites. However, key differences between the control and patient lines are observed. The ANK2-24 patient cell line shows clusters of grouped neurons, while the control line displays individualized neurons. Additionally, the ANK2-24 cell line exhibits more neuronal projections and dendrites compared to the control iPSC line.

#### Expression of ANK2 Protein

**Fig. 6:**
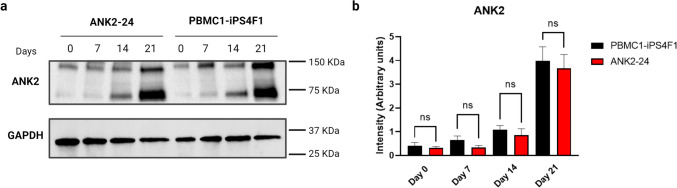
Expression of ANK2 protein. **a** Western blot analysis detecting the ANK2 protein in the PBMC1-iPS4F1 and ANK2-24 lines. GAPDH is used as a loading control. Molecular weights: ANK2 (135 kDa and 75 KDa) and GAPDH (36 kDa). **b** Analysis of ANK2 expression level. The PBMC1-iPS4F1 line shows a higher expression of the ANK2 protein than the ANK2-24 cell line. One-way ANOVA statistical analysis and Sidak’s multiple comparisons test. No significant differences (*p* > 0.05)

We confirmed ANK2 protein expression by western blot using an ankyrin B polyclonal antibody. We detected the expression of the ANK2 protein (135 KDa) in the PBMC1-iPS4F1 and the ANK2-24 cell lines according to their expected molecular weight. Moreover, we identified a band of 75 KDa in the control and patient lines corresponding to an ANK2 isoform produced by alternative splicing. Similarly, it occurs in the human cell line U-251MG (glioblastoma cell line) tested by the commercial supplier of the ankyrin B polyclonal antibody used in this study (Fig. 6a). On day 21 of neuronal differentiation, we detected a higher expression of ANK2 protein in both cell lines. The ANK2-24 cell line showed a decrease in ANK2 expression level compared to the control cell line from day 7 to day 21 of neuronal differentiation, but no significant differences were observed (Fig. 6b). Therefore, other regulatory factors of ANK2 expression could explain this decrease.

## Discussion

Tinnitus is a complex condition that affects a wide range of the population and is believed to have a genetic basis involving both common and rare variants [16]. Animal models, such as those induced by noise or ototoxic drugs, have been developed to study tinnitus [17–19], their association with environmentally induced hearing loss limits their ability to explore genetic contributions. To overcome this, an international effort is underway to develop non-animal models that reduce and refine the use of animals in biomedical research [20]. Personalized cellular models are emerging as essential tools for studying genetic variants in tinnitus neurobiology and conducting drug screening [21].

We generated a hiPSC line derived from a patient with severe tinnitus carrying a missense mutation in the *ANK2* gene (4:114,294,537 G/A) using a non-integrating Sendai virus method for reprogramming. This hiPSC line, characterized at both cellular and functional levels, demonstrates pluripotency and the ability to differentiate in vitro into the three germ layers of an embryo. These findings highlight the potential of developing hiPSC-based cellular models for studying tinnitus and screening drugs to restore the cellular phenotype.

The *ANK2* gene encodes ankyrin-B, a key protein involved in cellular functions such as motility, activation, proliferation, and maintaining specialized membrane domains. It has been extensively studied in autism spectrum disorder and is also implicated in cardiac and neurological diseases [11, 22, 23]. Exome sequencing in tinnitus patients has identified rare variants in *ANK2* associated with severe tinnitus [8, 9]. Although animal models have explored *ANK2*’s role in acoustic trauma and neuronal dysfunction [18, 22, 24], its involvement in auditory-evoked responses and neural development, particularly in tinnitus, has not been studied in human cellular models. In this study, we generated a cellular model derived from patients with MD and tinnitus to explore the role of ANK2 in auditory-evoked responses and neural development, comparing it with a control cell line.

The hiPSC-derived tinnitus model successfully differentiates into inner ear neurons within 21 days. Gene expression analysis using qPCR revealed differences in otic neuronal progenitor (ONP) formation between the control and patient lines, suggesting that the impaired *ANK2* gene function impacts the patient line.

In mice, the *Ank2* gene plays a critical role in maintaining pre-myelinated axons during early neurodevelopment. Loss-of-function mutants exhibit dysregulated calcium homeostasis and abnormal axonal branching [25]. A 2022 study showed high **Ank2** expression in the cerebral cortex during early neurodevelopment, regulating neural stem cell differentiation. Loss of *Ank2* alters neural development gene expression, contributing to an increased risk of ASD [22]. The study also highlights the importance of Pax2, Pax8, and FoxG1 in mouse inner ear development and auditory system differentiation. In a human cellular model, decreased ANK2 expression is associated with altered PAX8 and FOXG1 expression during neuronal differentiation [26–28]. However, further studies using additional patient-derived cell lines are needed to confirm these patterns. Despite a heterozygous benign mutation in the ANK2-24 cell line, β-III TUBULIN expression remained normal during neuronal differentiation that maturation into neurons was unaffected compared to the control line.

This study compared the formation and maturation of IENs between the control and patient lines, focusing on the expression of the transcription factor POU4F1, which plays a role in the formation and development of inner ear sensory neurons and neurite projections [29, 30]. The patient line showed increased neural projections and dendrites, which may be linked to hearing loss and tinnitus. Auditory sensitivity and tinnitus are interconnected, impacting quality of life. Increased neural activity, often triggered by early noise exposure, correlates with heightened sensitivity to auditory input and tinnitus perception [31–34]. The brain adapts by reorganizing neural connections, shaping how individuals with hearing loss and tinnitus perceive sound. Tinnitus involves heightened neural projections, which can contribute to emotional responses, and traumatic events may trigger its onset [32, 35, 36]. Understanding these changes is critical for developing targeted treatments for tinnitus, requiring an approach that addresses both auditory and neural factors [37–40].

It is important to recognize that the functional effects of hearing loss and tinnitus may vary between individuals, and that the relationship between neural changes and these conditions is complex. More research is needed to fully understand these dynamics and develop personalized treatments.

A major gap in understanding is how rare variants in the ANK2 gene contribute to severe tinnitus or inner ear neuron development in MD patients. Developing this hiPSC-based model with an ANK2 mutation is a crucial first step. This model provides a valuable resource for studying the mutation’s impact during neurodevelopment in MD.

hiPSC models are valuable tools for personalized medicine. They can be used to generate organoids and rebuild organs. Moreover, they can be used to screen drugs and test their efficacy. Additionally, gene therapy holds potential for reversing mutations and studying how they affect cellular phenotypes and functions [41–43].

## Conclusions

We have developed a hiPSC cell model able to differentiate into ONPs and IENs. This cell model serves as a valuable resource for drug screening and for testing gene therapies aimed at correcting the underlying mutation. The generation of this hiPSC model opens up promising opportunities for targeted and personalized interventions in treating severe tinnitus.

## Supplementary Information

Below is the link to the electronic supplementary material.

Supplementary file1 (DOCX 1099 KB)

Supplementary file2 (ZIP 4849 KB)

## Data Availability

No datasets were generated or analysed during the current study.
