# Supplementary material for: A Neuron-Like Cellular Model for Severe Tinnitus Associated with Rare Variations in the ANK2 Gene
Source: Mol Neurobiol. 2025 Jan 15;62(5):6467–77. doi: 10.1007/s12035-024-04674-8 (PMC11953095; doi:10.1007/s12035-024-04674-8)
Supplement: Supplementary file 1 — Supplementary file1 (DOCX 1099 KB) [file 12035_2024_4674_MOESM1_ESM.docx]

Molecular Neurobiology

A neuron-like cellular model for severe tinnitus associated with rare variations in the *ANK2* gene

Mar Lamolda^1,2^, Lidia Frejo^1,2,3*^, Juan Martin-Lagos^1,2,4^, Francisca E. Cara^1^, Alvaro Gallego-Martinez^1,2^, and Jose A. Lopez-Escamez^1,2,3^

^1^Division of Otolaryngology, Department of Surgery, Instituto de Investigación Biosanitaria, ibs.GRANADA, Granada, Universidad de Granada, Granada, Spain.

^2^Sensorineural Pathology Programme, Centro de Investigación Biomédica en Red en Enfermedades Raras, CIBERER, Madrid, Spain

^3^Meniere's Disease Neuroscience Research Program, Faculty of Medicine & Health, School of Medical Sciences, The Kolling Institute, University of Sydney, Sydney, New South Wales, Australia

^4^Department of Otorhinolaryngology, Hospital Clinico Universitario San Cecilio, Granada, Spain

**Corresponding Author:*

Lidia Frejo, PhD

*Email: lidia.frejonavarro@sydney.edu.au*

Supplementary Table 1. Primers

| Primer | 5’-3’ Fw | 5’-3’ Rv |
| --- | --- | --- |
| ANK2 | CTGAGAAATGGTGCCCTTGTTG | ATGTTGTAGAAGCAGCTGGACA |
| PAX8 | GAACCCTACCATGTTTGCCTG | TAGGGAGGTTGAATGGTTGCTG |
| FOXG1 | AAGAACTTCCCTTACTACCGCG | CTTCACGAAGCACTTGTTGAGG |
| β-III-TUBULIN | CATGGACGAGATGGAGTTCACC | CTTCGTACATCTCGCCCTCTTC |
| GAPDH | GACAACTTTGGTATCGTGGAAG | CAGTAGAGGCAGGGATGATGTT |

Supplementary Table 2. Antibodies

| Antibodies | Host | Dilution | Supplier | Ref |
| --- | --- | --- | --- | --- |
| ANK2 | rabbit | 1:50 | Invitrogen (Sweden) | PA5-82326 |
| PAX8 | mouse | 1:30 | Sigma Aldrich (USA) | SAB1404559 |
| FOXG1 | rabbit | 1:100 | Invitrogen (Taiwan) | PA5-117404 |
| POU4F1/BRNA3 | rabbit | 1:200 | Bioss (USA) | BS-3669R |
| β-III-TUBULIN | mouse | 1:100 | Invitrogen (Czech Republic) | MA1-19187 |
| Alexa Fluor 488 anti-rabbit IgG | goat | 1:700 | Invitrogen (USA) | A11008 |
| Alexa Fluor 555 anti-mouse IgG | donkey | 1:700 | Invitrogen (USA) | A31570 |
| GAPDH | chicken | 1:2000 | Sigma Aldrich (USA) | SAB3500247 |
| Anti-Chicken IgY (IgG) | rabbit | 1:2000 | Sigma Aldrich (USA) | A9046 |

**
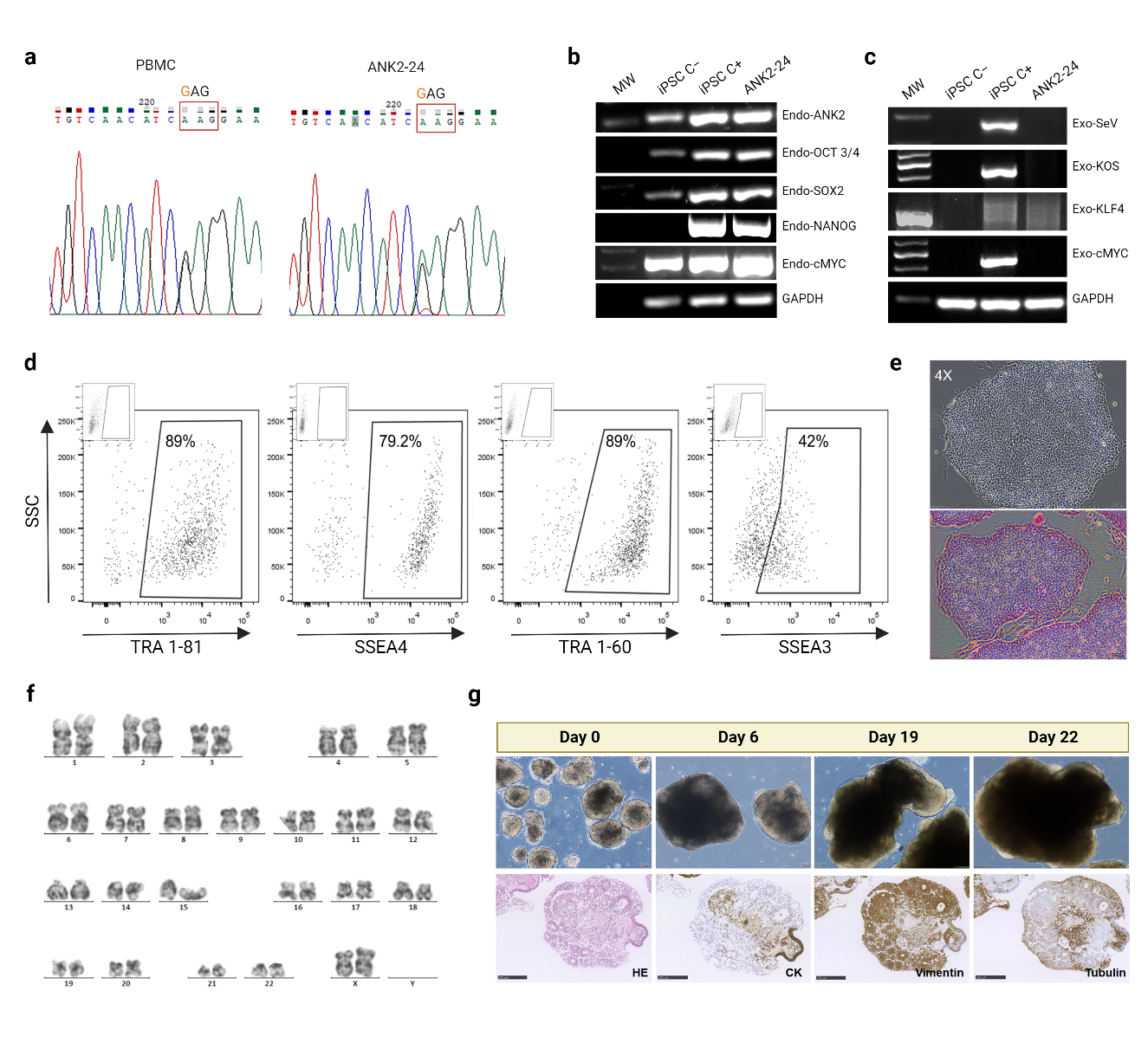
**

**Supplementary Fig.1** Cellular characterization of the ANK2-24 hiPSC line. a) Mutational analysis of the variant 4:114294537 G/A in *ANK2* gene from PBMCs (left panel) and hiPSC line, ANK2-24 (right panel). b) RT-PCR of the endogenous pluripotency genes *c-MYC, NANOG, SOX2,* and *OCT3/4*. c) RT-PCR analysis confirmed the silencing of exogenous reprogramming factors and SeV vector. PBMC transduced with SeV on day 4 were used as a positive control. Non-transduced PBMC from the same patient were used as a negative control. d) Expression of the pluripotency cell markers TRA1-81, SSEA4, TRA1-60 and SSEA3 by Flow Cytometry. e) Representative colonies of the ANK2-24 cell line were captured using an optical microscope (upper image). Alkaline phosphatase enzymatic activity staining was performed on ANK2-24 colonies (lower image). f) GTG-banding shows a normal karyotype (46, XX) in the ANK2-24 line. g) Embryo bodies (EB) differentiation assay for pluripotency. Immunohistochemistry analysis for endoderm (CKAE1-AE3), mesoderm (Vimentin), and ectoderm (β-III-Tubulin) from day 22 of EB formation
