# Supplementary figures and images for: A Neuron-Like Cellular Model for Severe Tinnitus Associated with Rare Variations in the ANK2 Gene
Source: Mol Neurobiol. 2025 Jan 15;62(5):6467–77. doi: 10.1007/s12035-024-04674-8 (PMC11953095; doi:10.1007/s12035-024-04674-8)

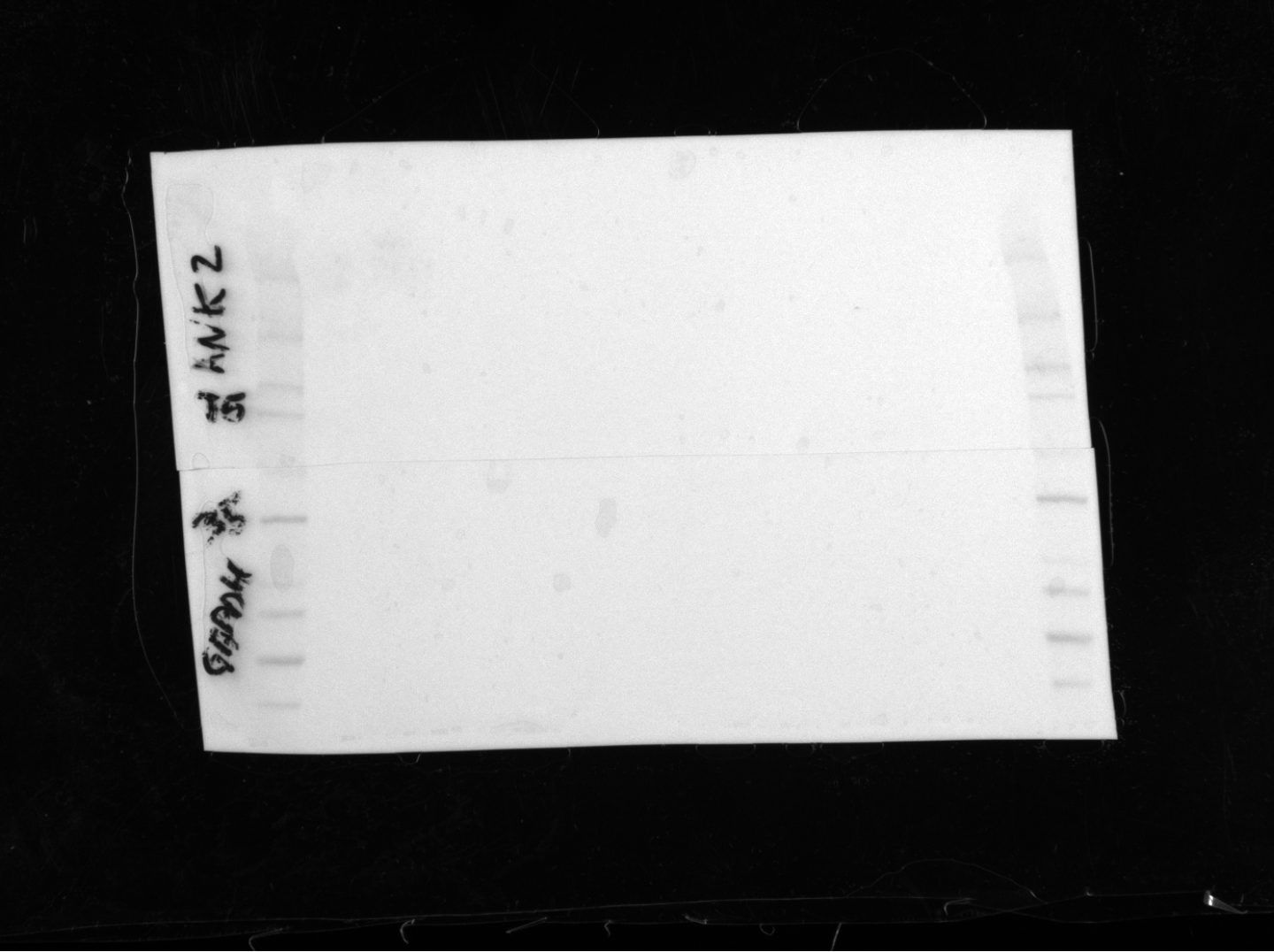

Supplement: Supplementary file 2 — Supplementary file2 (ZIP 4849 KB) [file 12035_2024_4674_MOESM2_ESM.zip › WesternoBlot_Membranes/1. Uncropped-Membrane_Colorimetric.tif]

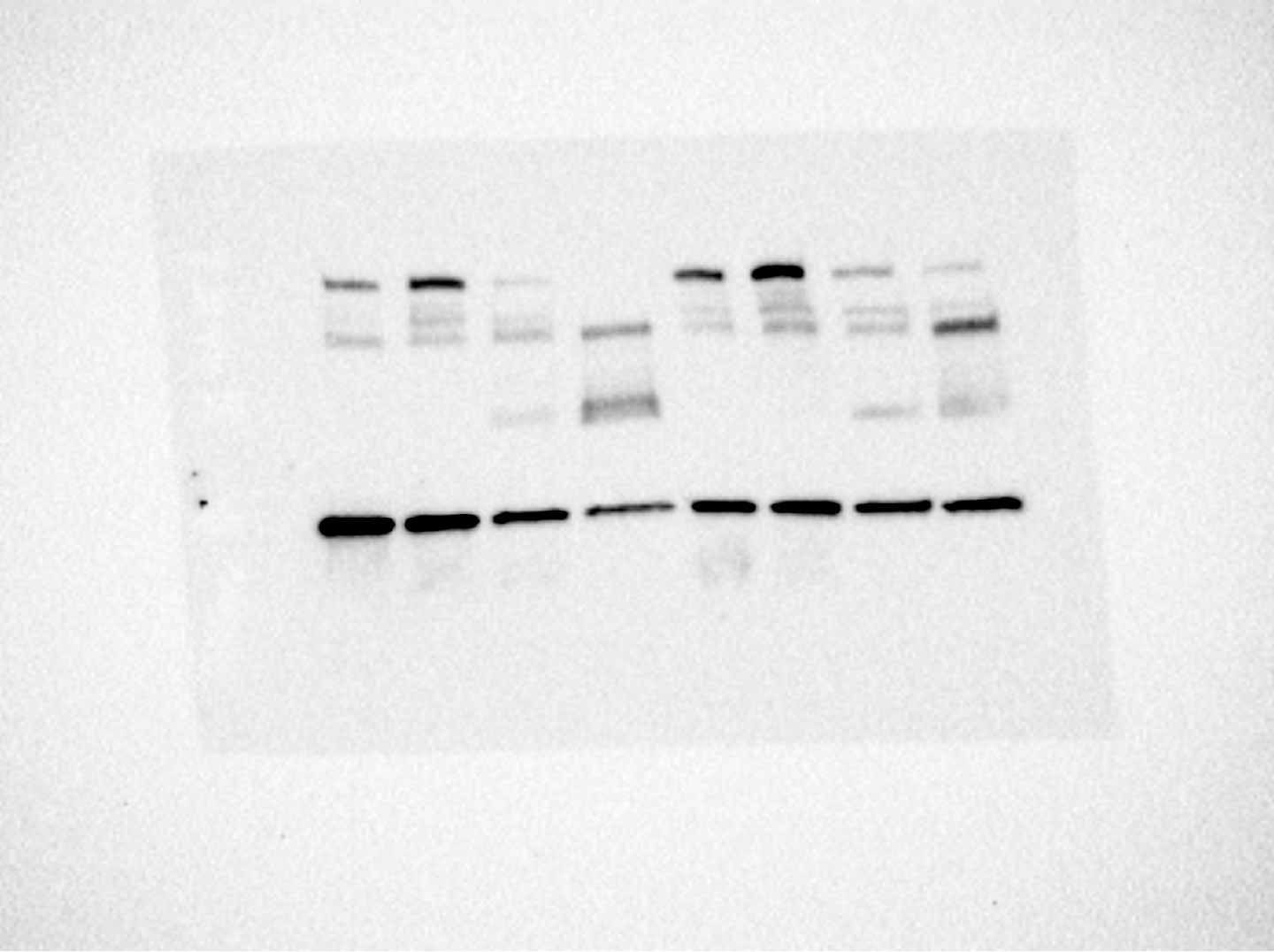

Supplement: Supplementary file 2 — Supplementary file2 (ZIP 4849 KB) [file 12035_2024_4674_MOESM2_ESM.zip › WesternoBlot_Membranes/2. Uncropped-Membrane.tif]

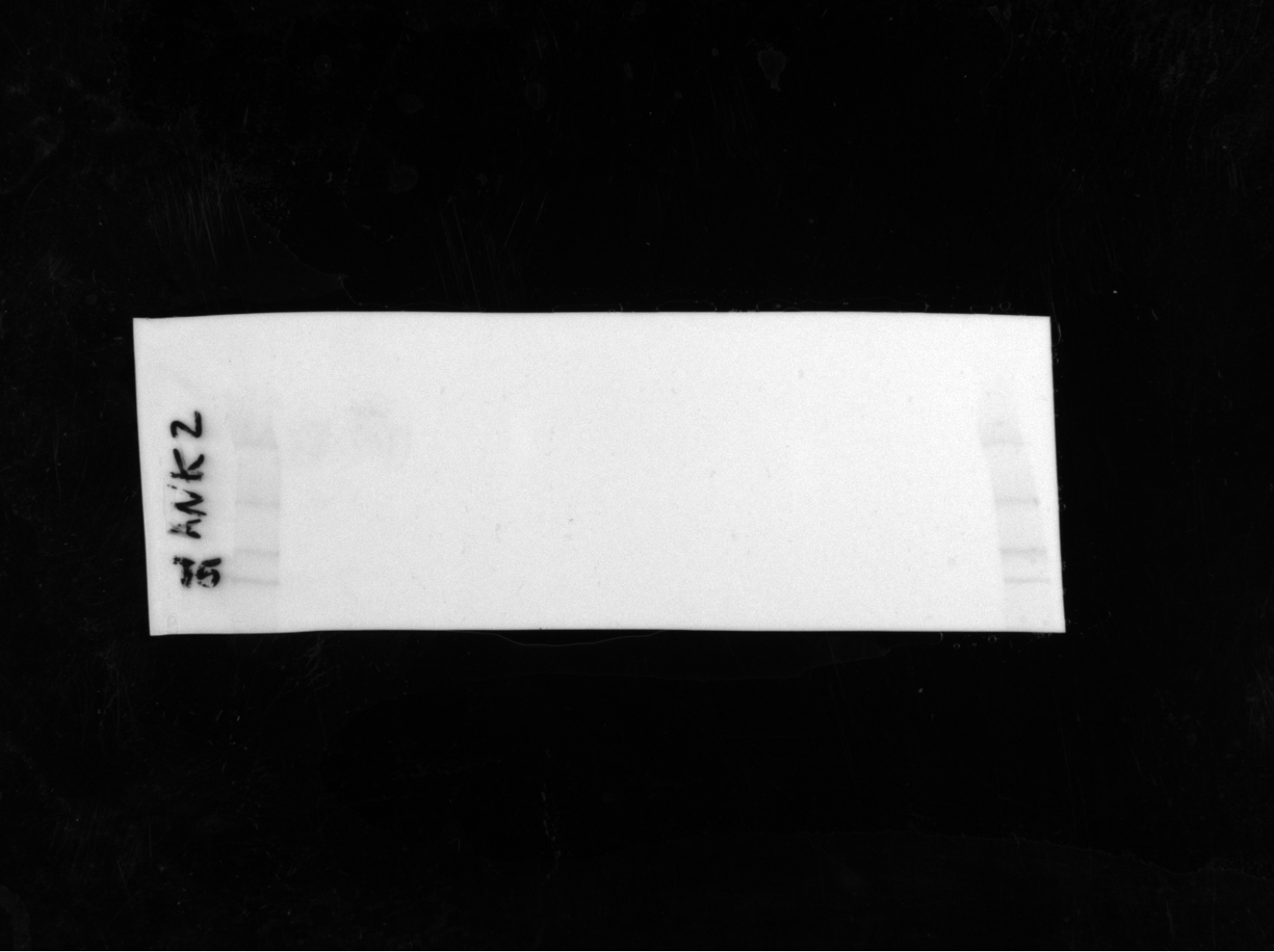

Supplement: Supplementary file 2 — Supplementary file2 (ZIP 4849 KB) [file 12035_2024_4674_MOESM2_ESM.zip › WesternoBlot_Membranes/3. ANK2_Colorimetric.tif]

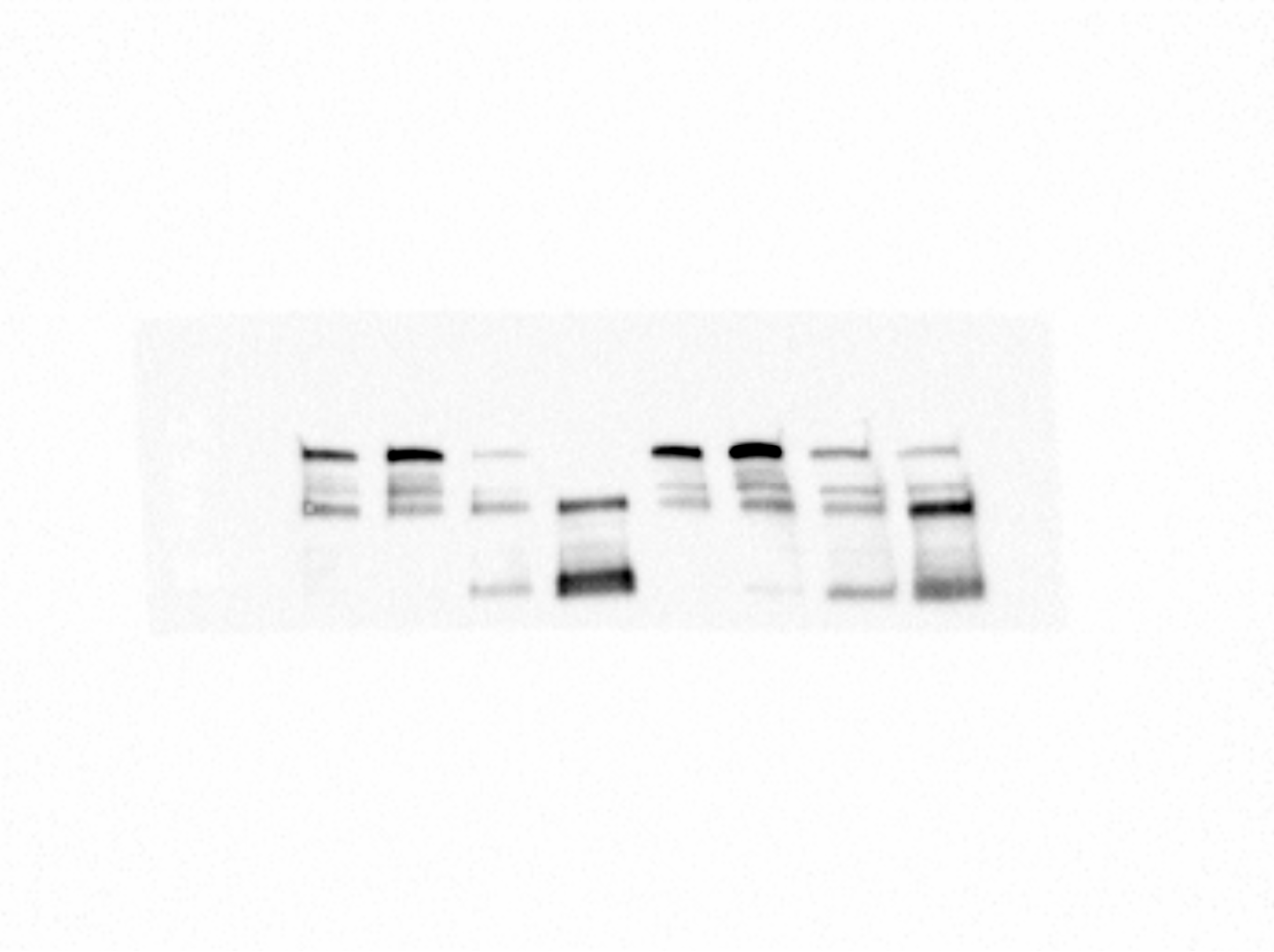

Supplement: Supplementary file 2 — Supplementary file2 (ZIP 4849 KB) [file 12035_2024_4674_MOESM2_ESM.zip › WesternoBlot_Membranes/4. ANK 2.tif]

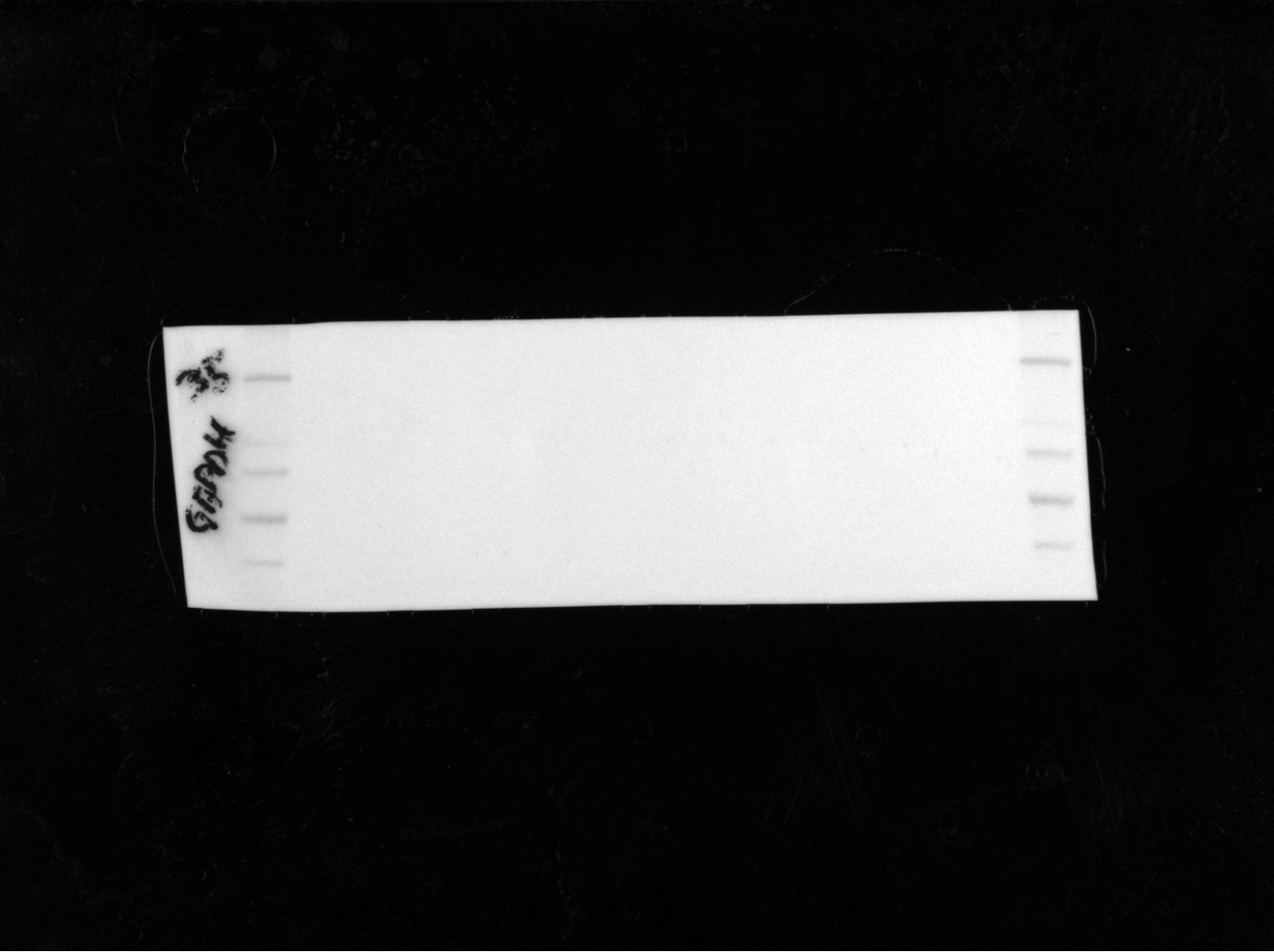

Supplement: Supplementary file 2 — Supplementary file2 (ZIP 4849 KB) [file 12035_2024_4674_MOESM2_ESM.zip › WesternoBlot_Membranes/5. GAPDH_Colorimetric.tif]

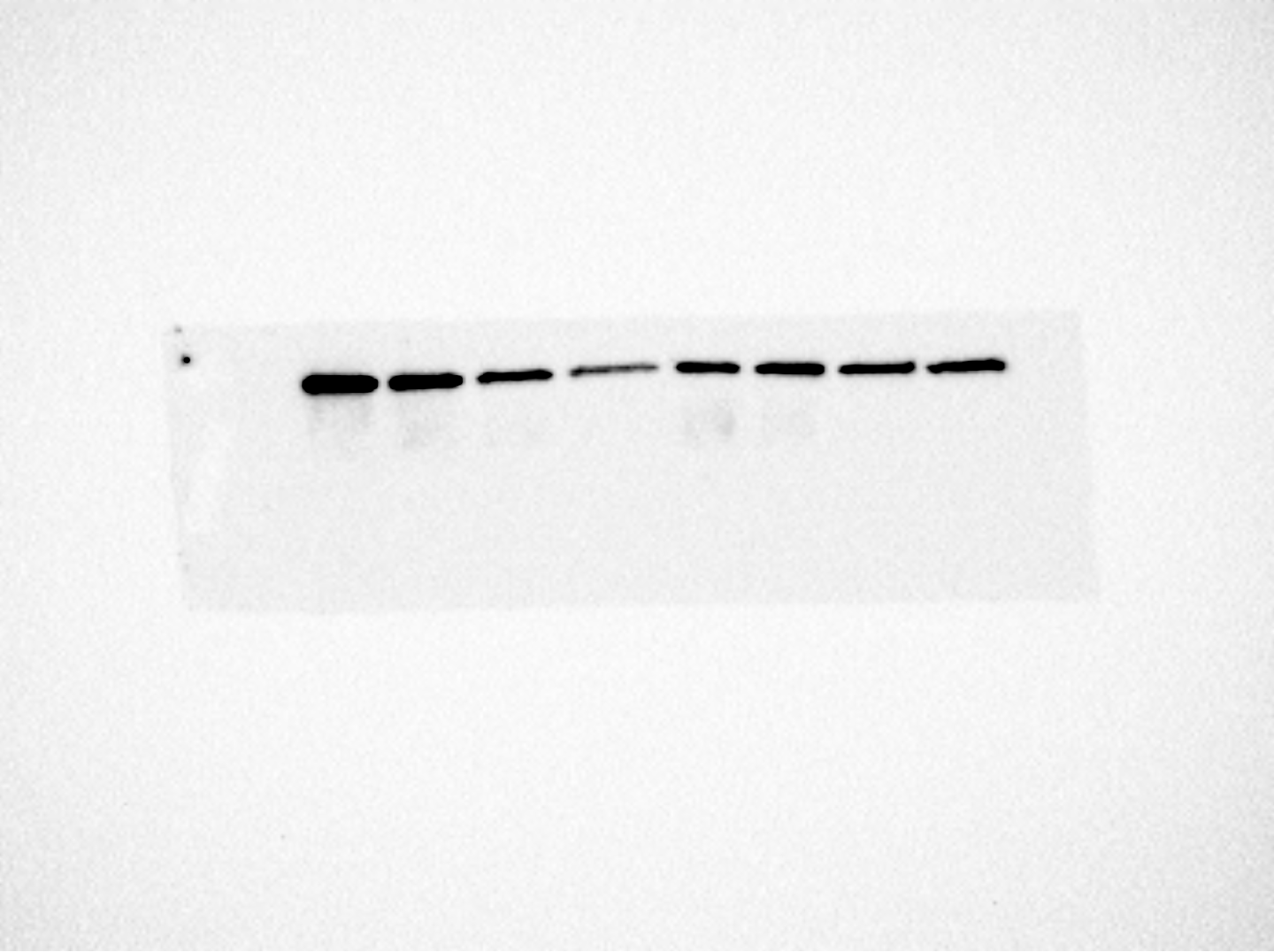

Supplement: Supplementary file 2 — Supplementary file2 (ZIP 4849 KB) [file 12035_2024_4674_MOESM2_ESM.zip › WesternoBlot_Membranes/6. GAPDH.tif]
